# The Hippo Pathway in Cardiac Regeneration and Homeostasis: New Perspectives for Cell-Free Therapy in the Injured Heart

**DOI:** 10.3390/biom10071024

**Published:** 2020-07-10

**Authors:** Mingjie Zheng, Joan Jacob, Shao-Hsi Hung, Jun Wang

**Affiliations:** 1 Department of Pediatrics, McGovern Medical School, The University of Texas Health Science Center at Houston, 6431 Fannin Street, Houston, TX 77030, USA; Mingjie.Zheng@uth.tmc.edu; 2 Graduate School of Biomedical Sciences, University of Texas MD Anderson Cancer Center and UTHealth, Houston, TX 77030, USA; Joan.Jacob@uth.tmc.edu (J.J.); Shao-Hsi.Hung@uth.tmc.edu (S.-H.H.)

**Keywords:** cardiac regeneration, Hippo pathway, proliferation, chromatin reprogramming, tissue homeostasis, immune response

## Abstract

Intractable cardiovascular diseases are leading causes of mortality around the world. Adult mammalian hearts have poor regenerative capacity and are not capable of self-repair after injury. Recent studies of cell-free therapeutics such as those designed to stimulate endogenous cardiac regeneration have uncovered new feasible therapeutic avenues for cardiac repair. The Hippo pathway, a fundamental pathway with pivotal roles in cell proliferation, survival and differentiation, has tremendous potential for therapeutic manipulation in cardiac regeneration. In this review, we summarize the most recent studies that have revealed the function of the Hippo pathway in heart regeneration and homeostasis. In particular, we discuss the molecular mechanisms of how the Hippo pathway maintains cardiac homeostasis by directing cardiomyocyte chromatin remodeling and regulating the cell-cell communication between cardiomyocytes and non-cardiomyocytes in the heart.

## 1. Introduction

Despite significant progress in the treatment of cardiovascular diseases, heart failure (HF) remains a predominant cause of death worldwide. Cardiac injury after myocardial infarction (MI) causes the irreversible damage of millions of cardiomyocytes, which induces inflammation and activates resting cardiac fibroblasts (CFs), and can eventually result in HF [1,2]. The adult zebrafish heart can regenerate through cardiomyocytes re-entering the cell-cycle and through revascularization in the injured region [3]. In contrast, the self-renewal capacity of cardiomyocytes in the adult mammalian heart is less than 1% per year, which has been a major hindrance to the self-repair of heart function after injury [4,5]. Heart transplantation is nearly the only option for the patients with end-stage HF [6]. Unfortunately, the number of heart donors is limited and does not cover the increasing demand for heart transplantation [7]. Furthermore, many HF patients are not eligible for heart transplantation because of concomitant comorbidities, and they have no other therapeutic options [8]. Therefore, developing novel therapeutics for treating the injured heart is essential.

Many cell-based and cell-free therapies have been proposed as a means for stimulating heart regeneration to protect the injured heart. Cell-based therapies were designed to replace dead cardiomyocytes and to reduce myocardial scar tissue by providing new sources of cardiomyocytes from the exogenous administration of progenitor cells including mesenchymal stem cells, bone marrow-derived cells and resident cardiac progenitor cells, as well as from the transplant of functional cardiomyocytes induced in vitro. Over the last two decades, considerable progress has been made in cell-based therapies, and many clinical trials have been conducted involving large cohorts of patients. Cell-based therapies are largely aimed to achieve therapeutic effects through two primary ways: the remuscularization of the injured area by exogenous healthy contractile cardiomyocytes and the activation of endogenous repair signaling through paracrine signaling [9,10]. However, these therapies have not led to significant improvements in cardiac function and have not succeeded in transforming contemporary cardiovascular practices [9,11,12]. Many roadblocks remain in implementing cell-based therapeutics into clinical practice, including efficacy of the therapy.

Over the last decade, based on the cumulative progress on the molecular mechanisms for regeneration, investigators have developed an alternative method for use in regenerative medicine that does not require cell transplantation—cell-free therapy. This type of therapy involves activating endogenous repair mechanisms by using soluble factors, extracellular vesicles [13,14,15,16] and microRNAs [17,18]. Biological and clinical researchers have recently used cell-free therapy for the treatment of injured hearts. This exciting new strategy involves stimulating intrinsic signaling pathways in cardiomyocytes to promote cardiac self-renewal [19]. The Hippo pathway is one of the endogenous pathways that has shown tremendous potential for therapeutic manipulation in cardiac regeneration. Studies in recent years have shown that the Hippo pathway is not just a highly conserved fundamental signaling pathway required for the development [20,21,22,23,24], homeostasis [25,26,27,28,29,30,31], and regeneration of the heart [32,33,34,35], but also has great therapeutic potential for cardiac regeneration [23,36]. In this review, we summarize the current findings on the role of the Hippo pathway in heart homeostasis and regeneration.

## 2. Overview of the Hippo Pathway

**Figure 1:**
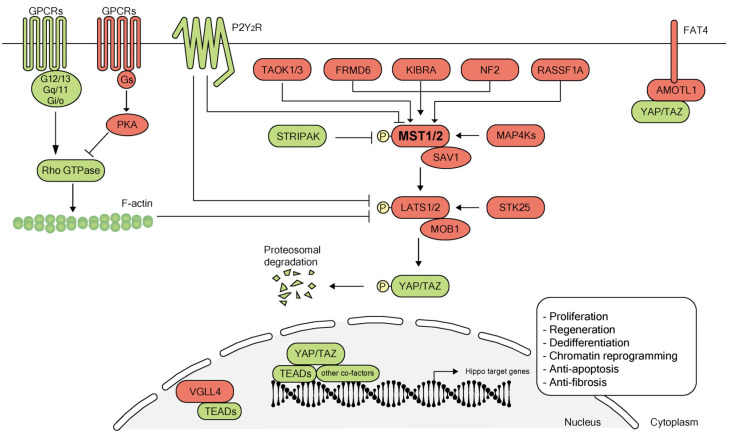
Overview of the Hippo pathway. The canonical Hippo signaling pathway is a complex network of proteins: mammalian sterile 20-like (MST) kinases 1/2, the adaptor proteins Salvador homolog 1 (SAV1), large tumor suppressor (LATS) kinases 1/2, Mps one binder kinase activator protein (MOB1), the downstream transcription cofactors Yes-associated protein 1 (YAP1) and its paralog transcriptional co-activator with PDZ-binding motif (TAZ, also known as WWTR1). When Hippo signaling is on (shown in red), kinases MST1/2 and SAV1 complex, phosphorylate and activate kinases LATS1/2. LATS1/2 interacts with the adaptor MOB1 to phosphorylate YAP and its analog TAZ. YAP/TAZ undergo ubiquitination and degradation after phosphorylation. When Hippo signaling is off (shown in green), YAP/TAZ as transcriptional co-activators translocate into the nucleus and interact with TEA domain family members (TEADs) and other co-factors such as SMAD family members (SMADs), β-Catenin, pituitary homeobox 2 (PITX2), forkhead box protein O1 (FOXO1), T-box transcription factor TBX5 and Runt-related transcription factor 1/2 (RUNX1/2) to regulate gene transcription for cellular proliferation, regeneration, dedifferentiation, apoptosis and chromatin reprogramming. Transcription cofactor vestigial-like protein 4 (VGLL4) competes directly with YAP for the binding of TEADs in the nucleus [56,57,70]. Hippo pathway activity also can be regulated by several upstream proteins such as kidney and brain expressed protein (KIBRA), neurofibromin 2 (NF2), FERM domain-containing protein 6 (FRMD6), mitogen-activated protein kinase kinase kinase kinases (MAP4Ks), striatin-interacting phosphatases and kinases (STRIPAK), serine/threonine-protein kinase 25 (STK25), Ras-associated domain family 1 isoform A (RASSF1A), thousand-and-one amino acid kinases 1/3 (TAOK1/3), P2Y_2_ nucleotide receptor (P2Y_2_R), protocadherin Fat4 (FAT4) and G-protein-coupled receptor signaling (GPCR). PKA, protein kinase A; AMOTL1, angiomotin-like protein 1.

The Hippo pathway is an evolutionarily conserved pathway that plays a critical role in regulating organ size and tissue homeostasis through controlling cell growth, proliferation, survival and differentiation [37,38]. The STE20 protein kinase family member Hippo was first studied in *Drosophila melanogaster*, where a mutation in the gene encoding Hippo resulted in grossly enlarged tissue and organ size [39]. Subsequently, other components of the canonical Hippo pathway were also delineated in *Drosophila* [40]. This central Hippo signaling kinase cascade in *Drosophila* is conserved in mammals. Mammalian Hippo ortholog STE20-like protein kinases (MST1/2) phosphorylate the large tumor suppressor homolog (LATS1/2). The MST1/2 kinases act together with their cofactor Salvador homolog 1 (SAV1) to phosphorylate LATS1/2 and their regulatory protein MOB domain kinase activator 1 (MOB1A/B). Activated LATS1 and LATS2 kinases subsequently phosphorylate the Hippo signaling downstream effector transcriptional co-activator protein Yorkie ortholog, Yes-associated protein (YAP)/transcriptional co-activator with PDZ-binding motif (TAZ), and inhibit the translocation of YAP/TAZ into the nucleus (Figure 1). When Hippo signaling is off, YAP/TAZ localizes and accumulates in the nucleus where it associates and binds to different transcription factors such as TEA domain transcription factor family members (TEADs) to enable transcription of downstream genes involved in different cellular processes (Figure 1). In addition to TEADs, studies have identified many other YAP/TAZ co-factors such as SMAD family members (SMADs) [41,42,43,44,45,46], β-Catenin [20], pituitary homeobox 2 (PITX2) [47], forkhead box protein O1 (FOXO1) [48], T-box transcription factor TBX5 [49,50], Runt-related transcription factor 1/2 (RUNX1/2) [51,52,53,54] and YAP/TAZ competing factors such as vestigial-like family member 4 (VGLL4) [55,56,57] (Figure 1). Hippo signaling activity can be regulated by variable upstream factors like kidney and brain expressed protein (KIBRA) [58], neurofibromin 2 (NF2) [58], FERM domain-containing protein 6 (FRMD6) [59], mitogen-activated protein kinase kinase kinase kinases (MAP4Ks) [60], striatin-interacting phosphatases and kinases (STRIPAK) [61], serine/threonine-protein kinase 25 (STK25) [62], P2Y_2_ nucleotide receptor (P2Y_2_R) [63], thousand-and-one amino acid kinases 1/3 (TAOK1/3) [64,65,66] and protocadherin Fat4 (FAT4) [67]. GPCRs (G-protein-coupled receptors) regulate Hippo signaling via GPCRs-G-protein-cytoskeleton axis [68,69] (Figure 1). In recent years, the Hippo-YAP pathway has become increasingly important as a potential endogenous mechanism for promoting organ regeneration.

## 3. Hippo Signaling Pathway in Heart Regeneration

The adult mammalian heart has a very limited capacity for regeneration. However, studies have shown the possibility of stimulating endogenous cardiac regeneration by manipulating various signals and pathways [19], but the intrinsic mechanisms of signals regulating heart regeneration are not well understood. Repression of Hippo signaling has promoted the proliferation of cardiomyocytes and controlled heart size during heart development [20]. Notably, manipulation of the Hippo-YAP pathway has shown great potential in stimulating endogenous cardiac regeneration [23,33,34,36]. Here, we briefly summarize studies that have illuminated the function of the Hippo pathway in cardiac regeneration and homeostasis, and we specifically focus on the progresses that have been made in the last two years.

### 3.1. The Hippo Pathway Regulates Cardiomyocyte Proliferation during Heart Regeneration

Heallen et al. conditionally deleted core Hippo signaling genes, *Sav1, Mst1*/*2* or *Lats2*, in the embryonic mouse heart by using the cardiac specific Nkx2-5-Cre driver, allowing cardiac specific deletion around embryonic day (E)7.5 [71]. They found that inactivation of Hippo signaling increased cardiomyocyte proliferation and led to overgrowth of the heart [20]. In contrast, Xin et al. found that cardiac-specific inactivation of Yap in mouse hearts using Nkx2-5-Cre driver substantially reduced cardiomyocyte proliferation and resulted in hearts with thinned myocardial layers, these Yap-deficient embryos died at E10.5 [21]. Von Gise et al. specifically deleted *Yap* in the heart using Tnnt2-Cre driver at E12.5, the embryos died at day E16.5 with severe hypoplasia in the myocardium [22]. In a recent study, Gan et al. found that the Lats1 and Lats2 kinases can directly phosphorylate the mTOR binding protein Raptor on Ser 606 [72]. They produced knock-in mice with the Raptor mutation of Ser^606^ to Asp, which mimics phosphorylation of Raptor. Compared with control mice, the *Raptor*^D/D^ knock-in mice had smaller hearts with reduced cell proliferation and size [72].

The regenerative ability of cardiomyocytes in mammalian hearts after birth is largely lost during postnatal development. During Postnatal Days 1–7, cardiomyocytes in the neonatal mouse heart are still capable of proliferating for substantial cardiac regeneration, but the ability is lost after Postnatal Day 7 [73,74]. Hippo signaling has been indicated to inhibit cardiomyocyte proliferation, thereby limiting cardiac regeneration in the adult heart. To examine the role of the Hippo signaling pathway in heart regeneration, Heallen et al. deleted Hippo signaling genes (*Salv* and *Lats1, 2*) specifically in mouse cardiomyocytes by using *Myh6^CreERT2^* transgene [33]. The mice with repressed Hippo signaling showed cardiomyocyte proliferation and cardiac regenerative capacity after injury at both Postnatal Day 8 and the adult stage, when the mouse heart has lost its regenerative capacity [33]. Notably, Leach et al. observed that the effects of established ischemic HF after MI can be reversed in *Myh6^CreERT2^;Salv^flox/flox^* mice, which had a smaller scar size and increased cardiac function compared with control hearts [36]. They used an adeno-associated virus 9 (AAV9) to deliver encoding short hairpin RNA (shRNA) against *Salv* in mice and found that these mice showed increased cardiomyocyte proliferation and enhanced heart function after the injection of AAV9-*Salv*-shRNA [36]. Serine 127 of human YAP is a critical site that is phosphorylated by LATS [75,76,77], YAP activity will increase after mutating serine 127 to alanine (S127A) [78,79]. In another study, constitutively activated human YAP (hYAPS127A) in mouse cardiomyocytes delivered by using AAV9 promoted cardiomyocyte division and proliferation and was sufficient to reduce scar size and increase cardiac function after MI [34]. Moreover, the cardiomyocyte-specific overexpression of constitutively active mouse YAP, mYAPS112A, enhanced heart regeneration during the nonregenerative stage in mice [23]. Previous studies investigating zebrafish heart regeneration have revealed that cardiomyocytes in adult zebrafish heart can re-enter the cell cycle and proliferate to promote regeneration after cardiac damage [3,80,81,82,83,84,85]. Lai et al. found that deletion of *wwtr1* in zebrafish lead to cardiac trabeculation reducing [86]. They also observed abnormal cell–cell junctions and amorphous cortical actin structure in cardiomyocytes in *wwtr1*-deficient zebrafish heart [86]. This study suggests that the Hippo pathway effector Wwtr1 is required for zebrafish cardiac trabeculation. In a recent study, Flinn et al. deleted the other core Hippo signaling effector gene *yap* in zebrafish [35]. Surprisingly, they found that not like in mouse, cardiomyocyte proliferation was not decreased in *yap^−^*^/*−*^ regenerating adult zebrafish hearts after cryoinjury [35]. However, significantly reduced collagen deposition was observed in adult yap mutant hearts at seven days post-injury. These results suggest that Yap is important for scar formation but not myocyte proliferation during adult zebrafish heart regeneration [35].

Mechanical stress is increasingly considered to be a key regulator of cell behavior directly related to cardiac physiology. YAP and TAZ are critical sensors of mechanical stress in several contexts [87,88,89]. In normal adult cardiomyocytes, YAP is absent in the nucleus but is found in the nucleus of cardiomyocytes in infarcted cardiac tissue with a stiffer extracellular matrix (ECM) [90]. The dystrophin-glycoprotein complex (DGC) links the actin cytoskeleton to ECM, and its deficiency results in muscular dystrophy, which is usually associated with dilated cardiomyopathy. Morikawa et al. revealed that the DGC component dystroglycan 1 (DAG1) interacts with phosphorylated Yap, inhibiting the nuclear location of Yap and decreasing cardiomyocyte proliferation [91]. Another group independently reported that the neonatal extracellular protein agrin promotes Yap localization in the nucleus and cardiomyocyte proliferation by interacting with the DGC and disrupting Yap–DGC complex formation. Agrin treatment inhibited fibrosis, regulated angiogenesis and the immune response, reduced infarct scar size and increased heart function after MI in mice. These results indicate that agrin treatment may have the potential to heal injured hearts [92]. Together, these studies suggest that Hippo signaling deficiency or YAP activation can stimulate cardiomyocyte proliferation to promote heart regeneration.

### 3.2. The Hippo Pathway and Reprogramming of Chromatin Accessibility during Heart Regeneration

Chromatin dynamics are inherent to cell reprogramming and cell fate transitions [93]. Chromatin opening is essential for the binding of transcriptional factors and the consequential transcriptional activation [94]. Cardiomyocytes are terminally differentiated cells, in which the chromatin state is stable and irreversible. However, they undergo chromatin remodeling and transient transcriptional reprogramming during regeneration [95,96,97,98]. During zebrafish heart regeneration, atrial cardiomyocytes can transdifferentiate into ventricular cardiomyocytes through cardiac reprogramming [98]. Zhang et al. used in vivo time-lapse imaging and found that Notch signaling is activated in zebrafish atrial endocardium following ventricular ablation, and discovered that blocking Notch signaling repressed the atrial-to-ventricular transdifferentiation and inhibited cardiac regeneration [98]. Previous exciting work revealed that the Hippo pathway can promote cellular reprogramming during regeneration of the intestine [99] and liver [100].

Despite the poor renewal ability of cardiomyocytes, YAP activation tends to promote cardiomyocyte proliferation and reduce infarct size in injured hearts in the adult mouse [34]. Recently, Monroe et al. generated mice that conditionally overexpressed YAP5SA in adult cardiomyocytes, which is a mutated YAP in which all LATS1/2 phosphorylation sites are changed from S to A [101]. By quantifying EdU-labeled cells, they reported that YAP5SA cardiomyocytes re-entered the cell cycle [101]. YAP5SA-overexpressing hearts had improved left ventricular muscle volume, increased numbers of left ventricular cardiomyocytes and decreased left ventricular chamber volume compared with controls, suggesting that YAP5SA promotes the generation of new cardiomyocytes [101]. In addition, they found that YAP5SA overexpression promoted cardiomyocyte proliferation with increased mononuclear cells without increased ploidy. Several studies have shown that increased numbers of mononuclear cardiomyocytes translates into improved renewal capacity but that an increase in ploidy was harmful for mice and zebrafish cardiomyocyte regeneration [102,103,104].

To further elucidate the molecular mechanisms of YAP5SA in the heart, Monroe et al. performed cardiomyocyte-specific nuclear RNA-sequencing, ATAC-sequencing and chromatin conformation capture assays. YAP5SA in cardiomyocytes upregulated cell-cycle related genes such as *Ccnd1*, *Dbf4*, *Mki67*, *Anln*, *Prkci* and *Numbl* and pro-proliferative transcription factors, *Myc*, *E2f1* and *E2f2* [101]. In YAP5SA-overexpressing cardiomyocytes, chromatin accessibility was reorganized with newly accessible loci for the binding of myocyte enhancer factor 2A (MEF2A) and activator protein 1 (AP-1). Together, these results indicate YAP5SA reverses adult cardiomyocytes to a proliferative fetal-like state by increasing chromatin accessibility and upregulating expression of fetal genes [101]. Thus, manipulating the Hippo pathway may be a feasible approach for heart regeneration therapy.

### 3.3. Essential Role of the Hippo Pathway in Heart Homeostasis

The heart is a highly differentiated multicellular organ that needs to maintain homeostasis. The physiological function of the Hippo pathway in the heart remains unclarified. However, recent studies have indicated an essential role of the Hippo pathway in maintaining cardiac homeostasis [26,31,105,106,107]. In response to injury, resting CFs are induced into activated CFs and myofibroblasts [108]. After cardiac injury, activated CFs tend to promote formation of fibrotic scars with a stiffened myocardial matrix. Initially, ECM formation is a benefit of wound healing as a protective mechanism, but progressive fibrosis results in irreversible stiffening and the loss of contractile ability. In a previous study, Del Re et al. found that Rassf1A interacted with Mst1 and inhibited the expression of nuclear factor (NF)-κB and tumor necrosis factor-α (TNF-α) to prevent proliferation and promote apoptosis in CFs [105]. Rassf1A is an endogenous activator of Mst1 and has different functional outcomes in cardiomyocytes and CFs [105]. However, the exact physiological functions of Hippo signaling in adult resting CFs is not well known.

Recent studies revealed that the development of CFs from epicardial progenitors was inhibited in *Lats1* and *Lats2* (*Lats1/2*)-deficient hearts [24]. Xiao et al. specifically deleted *Lats1/2* in embryonic cardiomyocytes by crossing mice with a *Lats1/2* conditional null allele with *Wt1^CreERT2^* mice. Using single-cell RNA sequencing, they reported that *Lats1/2* mutant CFs stayed in an intermediate cell state with both fibroblast and epicardial characteristics and failed to activate CF differentiation [24]. Using *Tcf21^iCre^; Lats1^fl/fl^; Lats2^fl/fl^* mice, Xiao et al. specifically deleted *Lats1/2* in CFs to examine the function of the Hippo signaling pathway in adult resting CFs under physiologic conditions [29]. In the subepicardial and subendocardial regions of *Tcf21^iCre^; Lats1^fl/fl^; Lats2^fl/fl^* mouse ventricles, they noted primary fibrosis that exhibited a self-perpetuating fibrotic response [29,109]. *Tcf21^iCre^; Lats1^fl/fl^; Lats2^fl/fl^* mice hearts showed reduced cardiac output and increased fractional shortening and ejection fraction after tamoxifen injection. These results suggest that the activation of cardiac fibrosis in adult resting CFs is spontaneous after *Lats1/2* deletion. Interestingly, similar to cells from recovered hearts after MI, they found increased heterogeneity of myeloid cells in uninjured *Lats1/2* deficient hearts [29]. Collectively, these studies indicate that *Lats1/2* may play an important role in maintaining the resting CF cell state by inhibiting the Yap-involved injury response.

Pressure overload causes cardiac hypertrophy and eventually leads to HF due to massive cardiomyocyte death and loss [110,111]. In a physiological study of the Hippo pathway, Ikeda et al. reported that activation of YAP induced cardiac dysfunction and cardiomyocyte dedifferentiation in the long-term in the presence of pressure overload [112]. They specifically deleted *Salv* in cardiomyocytes by using *Myh6^Cre^* mice and created a pressure overload model by transverse aortic constriction operation. In *Salv* deletion mice, Yap accumulated in the nucleus of cardiomyocytes. Salv-deficient mice exacerbated the progression of cardiac dysfunction and sarcomere disarray in cardiomyocytes induced by pressure overload with an activated cardiomyocyte cell cycle and decreased apoptosis. Moreover, cardiomyocyte dedifferentiation was attenuated in *Salv* deletion mice.

Diabetic cardiomyopathy can develop in HF patients with obesity and diabetes [113], accompanied by high blood pressure, reduced left ventricular ejection fraction (LVEF) and preserved LVEF [114]. In a type 2 diabetes mouse model, Ikeda et al. recently reported that the level of phospho-Lats2 was significantly downregulated, but Yap was activated in cardiomyocytes of diabetic mice after consumption of a high-fat diet for eight weeks. They also assessed YAP concentration in biopsy samples from humans with and without type 2 diabetes. Those studies showed that the concentration of YAP in the biopsy specimens from human showed a positive correlation to hemoglobin A1C level, which is commonly used to measure average blood sugar level for diabetes diagnosis. In addition, they used a small-molecule specific inhibitor, verteporfin [112], to investigate the interventions in their model. The inhibitor can disrupt the interaction between YAP and TEAD. The results show that verteporfin treatment significantly increased the survival rate after pressure overload in mice fed high-fat diets. In another study, Triastuti et al. used a novel inhibitor XMU-MP-1, which blocked the central Hippo kinase Mst1/2, to demonstrate that an increase in the Yap-complex protected the mouse heart against pressure overload [115].

### 3.4. The Hippo Pathway and Immune Responses during Heart Regeneration

The immune system plays a complicated, but key role in promoting both the regenerative response and the acute inflammatory response after cardiac injury [116]. The inflammatory response is a double-edged sword after injury, as it is both necessary and beneficial at the initial stages but deleterious at later stages of the injury response [117]. Recent studies revealed that the inflammatory response plays both constructive and harmful roles during heart regeneration [118,119,120]. The exact molecular mechanisms underlying the effects of the immune response in heart regeneration are still to be elucidated.

Molkentin and colleagues [121,122,123] worked with cardiac progenitor cells and bone marrow mononuclear cells, which are currently being tested in clinical trials of cardiac cell therapy. They found that mice injected with cardiac progenitor cells and bone marrow mononuclear cells showed significantly improved ventricular performance and cardiac function after ischemia–reperfusion injury. In addition, they surprisingly observed that injecting dead cells or a chemical inducer of the innate immune response called zymosan [124] also healed the injured heart. The exogenous cells induced an acute sterile immune response, which resulted in the spatiotemporal induction of macrophages that improved heart function [121]. This response of inducing macrophages in the infarct border zone altered CF activity, enhanced the mechanical properties in the injured area, and decreased the ECM in the border zone. An acute inflammatory response-based wound healing is beneficial in cardiac cell therapy that rejuvenates the heart after MI [121]. Although cell-based therapies have shown a great potential for cardiac regeneration, their success relies heavily on the innate immune response.

Ramjee et al. conditionally deleted the Hippo signaling downstream effector genes, *Yap* and *Taz*, in the mouse epicardium by using an inducible Wt1Cre driver [125]. They noted that mice with an epicardial deficiency of Yap and Taz had fewer regulatory T cells (Tregs) in the myocardium and decreased expression of interferon-γ, which is an inducer of Tregs. In several studies, Treg cells in pregnant mice suppressed inflammation, improved heart function, and even contributed to an increase in maternal heart size [126,127,128,129]. Thus, Tregs may play an essential role in regulating cardiac function, and Hippo signaling is important for epicardial cells by inducing Treg cells after injury [125].

In the resting heart, the primary immune cells are macrophages that are found in the interstitium among cardiomyocytes and surrounding endothelial cells [116,130]. Macrophages play a vital role in cardiac regeneration [117]. Ramjee et al. found that macrophages were significantly increased in the epicardium of *Yap/Taz*–mutant mice after injury [125]. In a recent study, Xiao et al. performed Drop-sequencing in control and *Tcf21^iCre^; Lats1^fl/fl^; Lats2^fl/fl^* mice to address if *Lats1/2*-null resting CFs promoted their differentiation or caused an injury response [29]. Clustering analysis of single-cell expression profiling data revealed eight monocyte/macrophage clusters (Mϕ1–8) and found that macrophages showed a proliferative state [131]. Using immunofluorescence staining with the myeloid marker Lyz, they confirmed increased numbers and composition of macrophages in *Tcf21^iCre^; Lats1^fl/fl^; Lats2^fl/fl^* mice hearts [29]. Furthermore, Xiao et al. found all macrophage clusters displayed high levels of myeloid lineage specific regulons that were not active in CFs by using Uniform Manifold Approximation and Projection (UMAP) [29]. Francisco et al. revealed that RASSF1A attenuates inflammatory cytokine expression and antagonizes ischemia/reperfusion-induced myocardial inflammation by negatively regulating the expression of NF-κB [132]. Collectively, these studies show that the Hippo pathway maintains the immunological balance in the heart and deficiency of Hippo signaling leads to a cardiac imbalance of macrophages.

## 4. Perspectives and Conclusions

**Figure 2:**
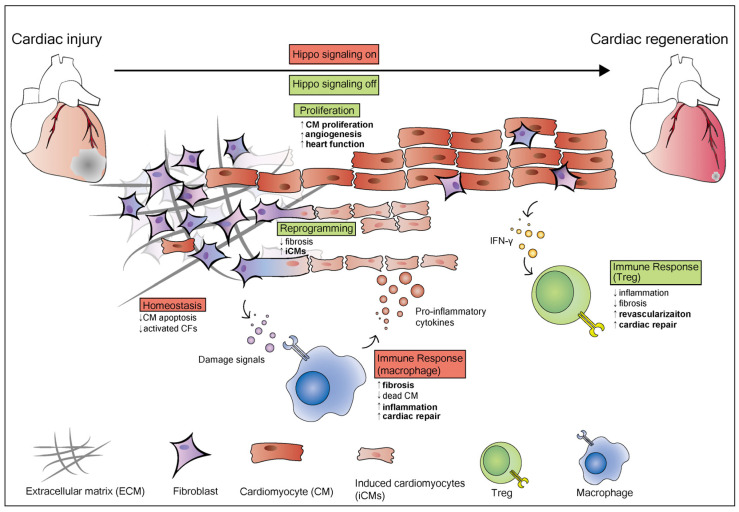
The role of the Hippo pathway in heart regeneration and homeostasis. The Hippo pathway plays different roles in different cardiac cell types. When Hippo signaling is on (shown in red), it maintains heart homeostasis and inhibits CM apoptosis and activated CFs. After an injury, the damaged area in the heart consists of dead cardiomyocytes, activated cardiac fibroblasts, extracellular matrix (ECM), and immune cells including macrophages and T-regulatory (Treg) cells. When the heart is injured, the homeostasis of the heart is destroyed. Damage signals induce an immune response that activates resident macrophages, which release pro-inflammatory cytokines to induce inflammation and fibrosis. Resting cardiac fibroblasts induced into activated CFs and myofibroblasts, then activate CFs which promotes fibrosis in the damaged area. Cardiac fibroblasts activation also can be achieved through inhibition of the Hippo pathway. In cardiomyocytes, Yap activation induces cardiomyocytes to re-enter the cell cycle and proliferate when Hippo signaling is off (shown in green). YAP5SA overexpression promotes the generation of new cardiomyocytes and reorganizes chromatin accessibility to a proliferative fetal-like state. In epicardial cells, the Hippo pathway promotes the secretion of cytokines such as interferon-γ (IFNγ), which recruits T-regulatory (Treg) cells. Treg cells inhibit inflammation and fibrosis, promote cardiac repair and revascularization.

In modern medicine, integrated methods of heart regeneration should include five hallmarks that Bertero and Murry proposed in 2018, including remuscularization, immunological balance, angiogenesis and arteriogenesis, resolution of fibrosis and electromechanical stability [133]. Above, the studies have shown that manipulating the Hippo-YAP pathway promotes remuscularization and angiogenesis during heart regeneration (Figure 2). Manipulating the Hippo-YAP pathway can also attenuate fibrosis development and facilitate resolution of fibrosis after MI [134]. In addition, the studies above indicate that the Hippo-YAP pathway may be a promising therapeutic target in diabetic patients with high blood pressure.

In conclusion, recent discoveries have unveiled novel functions of the Hippo pathway in regulating heart regeneration and cardiac homeostasis. Specifically, the Hippo pathway plays a unique role in regulating the proliferation, immune responses, chromatin reprogramming, cell state transitions and cell-to-cell communications in the heart. The combined results of high-throughput approaches, such as single-cell sequencing, ATAC-sequencing and ChIP-sequencing, and functional studies in animal models have revealed that the Hippo signaling pathway regulates epigenetics, transcriptomics, proteomics and cytologic changes to promote cardiomyocyte regeneration. Delicate manipulation of the Hippo-YAP signaling pathway has immense potential as a cell-free regenerative therapy for promoting adult heart renewal and treating heart disease [135]. Cardiomyocyte counts have been shown to increase by 40% after inducing YAP5SA overexpression and YAP5SA lineage cardiomyocytes coupled to pre-existing cardiomyocytes [101]. However, YAP1 has been shown to be an oncogene in various human cancers and promotes intense proliferation in cancer cells [136,137]. Current clinical trials of cardiac regenerative therapies have encountered obstacles, suggesting limitations and difficulties in translating preclinical studies of cardiac regeneration into clinical medicine. Nevertheless, targeting the Hippo-YAP signaling pathway is a promising cell-free therapeutic approach for cardiac regenerative medicine.

## References

[1] Souders C.A., Bowers S.L., Baudino T.A. (2009). Cardiac fibroblast: The renaissance cell. Circ. Res..

[2] Takeda N., Manabe I., Uchino Y., Eguchi K., Matsumoto S., Nishimura S., Shindo T., Sano M., Otsu K., Snider P. (2010). Cardiac fibroblasts are essential for the adaptive response of the murine heart to pressure overload. J. Clin. Investig..

[3] Poss K.D., Wilson L.G., Keating M.T. (2002). Heart regeneration in zebrafish. Science.

[4] Zhang Y., Mignone J., MacLellan W.R. (2015). Cardiac Regeneration and Stem Cells. Physiol. Rev..

[5] Senyo S.E., Steinhauser M.L., Pizzimenti C.L., Yang V.K., Cai L., Wang M., Wu T.D., Guerquin-Kern J.L., Lechene C.P., Lee R.T. (2013). Mammalian heart renewal by pre-existing cardiomyocytes. Nature.

[6] Wilhelm M.J. (2015). Long-term outcome following heart transplantation: Current perspective. J. Thorac. Dis..

[7] Stehlik J., Edwards L.B., Kucheryavaya A.Y., Benden C., Christie J.D., Dobbels F., Kirk R., Rahmel A.O., Hertz M.I. (2011). The Registry of the International Society for Heart and Lung Transplantation: Twenty-eighth Adult Heart Transplant Report—2011. J. Heart Lung Transplant..

[8] Lenzen M.J., Boersma E., Reimer W.J., Balk A.H., Komajda M., Swedberg K., Follath F., Jimenez-Navarro M., Simoons M.L., Cleland J.G. (2005). Under-utilization of evidence-based drug treatment in patients with heart failure is only partially explained by dissimilarity to patients enrolled in landmark trials: A report from the Euro Heart Survey on Heart Failure. Eur. Heart J..

[9] Menasche P. (2018). Cell therapy trials for heart regeneration—lessons learned and future directions. Nat. Rev. Cardiol..

[10] Nakamura K., Murry C.E. (2019). Function Follows Form—A Review of Cardiac Cell Therapy. Circ. J..

[11] Madonna R., Van Laake L.W., Davidson S.M., Engel F.B., Hausenloy D.J., Lecour S., Leor J., Perrino C., Schulz R., Ytrehus K. (2016). Position Paper of the European Society of Cardiology Working Group Cellular Biology of the Heart: Cell-based therapies for myocardial repair and regeneration in ischemic heart disease and heart failure. Eur. Heart J..

[12] Eschenhagen T., Bolli R., Braun T., Field L.J., Fleischmann B.K., Frisen J., Giacca M., Hare J.M., Houser S., Lee R.T. (2017). Cardiomyocyte Regeneration: A Consensus Statement. Circulation.

[13] Liu B., Lee B.W., Nakanishi K., Villasante A., Williamson R., Metz J., Kim J., Kanai M., Bi L., Brown K. (2018). Cardiac recovery via extended cell-free delivery of extracellular vesicles secreted by cardiomyocytes derived from induced pluripotent stem cells. Nat. Biomed. Eng..

[14] Tan S.J.O., Floriano J.F., Nicastro L., Emanueli C., Catapano F. (2020). Novel Applications of Mesenchymal Stem Cell-derived Exosomes for Myocardial Infarction Therapeutics. Biomolecules.

[15] Emanueli C., Shearn A.I., Angelini G.D., Sahoo S. (2015). Exosomes and exosomal miRNAs in cardiovascular protection and repair. Vasc. Pharmacol..

[16] Sahoo S., Losordo D.W. (2014). Exosomes and cardiac repair after myocardial infarction. Circ. Res..

[17] Verjans R., van Bilsen M., Schroen B. (2020). Reviewing the Limitations of Adult Mammalian Cardiac Regeneration: Noncoding RNAs as Regulators of Cardiomyogenesis. Biomolecules.

[18] Olson E.N. (2014). MicroRNAs as therapeutic targets and biomarkers of cardiovascular disease. Sci. Transl. Med..

[19] Heallen T.R., Kadow Z.A., Kim J.H., Wang J., Martin J.F. (2019). Stimulating Cardiogenesis as a Treatment for Heart Failure. Circ. Res..

[20] Heallen T., Zhang M., Wang J., Bonilla-Claudio M., Klysik E., Johnson R.L., Martin J.F. (2011). Hippo pathway inhibits Wnt signaling to restrain cardiomyocyte proliferation and heart size. Science.

[21] Xin M., Kim Y., Sutherland L.B., Qi X., McAnally J., Schwartz R.J., Richardson J.A., Bassel-Duby R., Olson E.N. (2011). Regulation of insulin-like growth factor signaling by Yap governs cardiomyocyte proliferation and embryonic heart size. Sci. Signal..

[22] Von Gise A., Lin Z., Schlegelmilch K., Honor L.B., Pan G.M., Buck J.N., Ma Q., Ishiwata T., Zhou B., Camargo F.D. (2012). YAP1, the nuclear target of Hippo signaling, stimulates heart growth through cardiomyocyte proliferation but not hypertrophy. Proc. Natl. Acad. Sci. USA.

[23] Xin M., Kim Y., Sutherland L.B., Murakami M., Qi X., McAnally J., Porrello E.R., Mahmoud A.I., Tan W., Shelton J.M. (2013). Hippo pathway effector Yap promotes cardiac regeneration. Proc. Natl. Acad. Sci. USA.

[24] Xiao Y., Hill M.C., Zhang M., Martin T.J., Morikawa Y., Wang S., Moise A.R., Wythe J.D., Martin J.F. (2018). Hippo Signaling Plays an Essential Role in Cell State Transitions during Cardiac Fibroblast Development. Dev. Cell.

[25] Yamamoto S., Yang G., Zablocki D., Liu J., Hong C., Kim S.J., Soler S., Odashima M., Thaisz J., Yehia G. (2003). Activation of Mst1 causes dilated cardiomyopathy by stimulating apoptosis without compensatory ventricular myocyte hypertrophy. J. Clin. Investig..

[26] Odashima M., Usui S., Takagi H., Hong C., Liu J., Yokota M., Sadoshima J. (2007). Inhibition of endogenous Mst1 prevents apoptosis and cardiac dysfunction without affecting cardiac hypertrophy after myocardial infarction. Circ. Res..

[27] Wang P., Mao B., Luo W., Wei B., Jiang W., Liu D., Song L., Ji G., Yang Z., Lai Y.Q. (2014). The alteration of Hippo/YAP signaling in the development of hypertrophic cardiomyopathy. Basic Res. Cardiol..

[28] Matsui Y., Nakano N., Shao D., Gao S., Luo W., Hong C., Zhai P., Holle E., Yu X., Yabuta N. (2008). Lats2 is a negative regulator of myocyte size in the heart. Circ. Res..

[29] Xiao Y., Hill M.C., Li L., Deshmukh V., Martin T.J., Wang J., Martin J.F. (2019). Hippo pathway deletion in adult resting cardiac fibroblasts initiates a cell state transition with spontaneous and self-sustaining fibrosis. Genes Dev..

[30] Yu W., Ma X., Xu J., Heumuller A.W., Fei Z., Feng X., Wang X., Liu K., Li J., Cui G. (2019). VGLL4 plays a critical role in heart valve development and homeostasis. PLoS Genet..

[31] Del Re D.P., Yang Y., Nakano N., Cho J., Zhai P., Yamamoto T., Zhang N., Yabuta N., Nojima H., Pan D. (2013). Yes-associated protein isoform 1 (Yap1) promotes cardiomyocyte survival and growth to protect against myocardial ischemic injury. J. Biol. Chem..

[32] Wang J., Liu S., Heallen T., Martin J.F. (2018). The Hippo pathway in the heart: Pivotal roles in development, disease, and regeneration. Nat. Rev. Cardiol..

[33] Heallen T., Morikawa Y., Leach J., Tao G., Willerson J.T., Johnson R.L., Martin J.F. (2013). Hippo signaling impedes adult heart regeneration. Development.

[34] Lin Z., von Gise A., Zhou P., Gu F., Ma Q., Jiang J., Yau A.L., Buck J.N., Gouin K.A., van Gorp P.R. (2014). Cardiac-specific YAP activation improves cardiac function and survival in an experimental murine MI model. Circ. Res..

[35] Flinn M.A., Jeffery B.E., O’Meara C.C., Link B.A. (2019). Yap is required for scar formation but not myocyte proliferation during heart regeneration in zebrafish. Cardiovasc. Res..

[36] Leach J.P., Heallen T., Zhang M., Rahmani M., Morikawa Y., Hill M.C., Segura A., Willerson J.T., Martin J.F. (2017). Hippo pathway deficiency reverses systolic heart failure after infarction. Nature.

[37] Pan D. (2010). The hippo signaling pathway in development and cancer. Dev. Cell.

[38] Zhao B., Tumaneng K., Guan K.L. (2011). The Hippo pathway in organ size control, tissue regeneration and stem cell self-renewal. Nat. Cell Biol..

[39] Jia J., Zhang W., Wang B., Trinko R., Jiang J. (2003). The Drosophila Ste20 family kinase dMST functions as a tumor suppressor by restricting cell proliferation and promoting apoptosis. Genes Dev..

[40] Halder G., Johnson R.L. (2011). Hippo signaling: Growth control and beyond. Development.

[41] Ferrigno O., Lallemand F., Verrecchia F., L’Hoste S., Camonis J., Atfi A., Mauviel A. (2002). Yes-associated protein (YAP65) interacts with Smad7 and potentiates its inhibitory activity against TGF-beta/Smad signaling. Oncogene.

[42] Varelas X., Sakuma R., Samavarchi-Tehrani P., Peerani R., Rao B.M., Dembowy J., Yaffe M.B., Zandstra P.W., Wrana J.L. (2008). TAZ controls Smad nucleocytoplasmic shuttling and regulates human embryonic stem-cell self-renewal. Nat. Cell Biol..

[43] Alarcon C., Zaromytidou A.I., Xi Q., Gao S., Yu J., Fujisawa S., Barlas A., Miller A.N., Manova-Todorova K., Macias M.J. (2009). Nuclear CDKs drive Smad transcriptional activation and turnover in BMP and TGF-beta pathways. Cell.

[44] Varelas X., Samavarchi-Tehrani P., Narimatsu M., Weiss A., Cockburn K., Larsen B.G., Rossant J., Wrana J.L. (2010). The Crumbs complex couples cell density sensing to Hippo-dependent control of the TGF-beta-SMAD pathway. Dev. Cell.

[45] Fujii M., Toyoda T., Nakanishi H., Yatabe Y., Sato A., Matsudaira Y., Ito H., Murakami H., Kondo Y., Kondo E. (2012). TGF-beta synergizes with defects in the Hippo pathway to stimulate human malignant mesothelioma growth. J. Exp. Med..

[46] Narimatsu M., Samavarchi-Tehrani P., Varelas X., Wrana J.L. (2015). Distinct polarity cues direct Taz/Yap and TGFbeta receptor localization to differentially control TGFbeta-induced Smad signaling. Dev. Cell.

[47] Tao G., Kahr P.C., Morikawa Y., Zhang M., Rahmani M., Heallen T.R., Li L., Sun Z., Olson E.N., Amendt B.A. (2016). Pitx2 promotes heart repair by activating the antioxidant response after cardiac injury. Nature.

[48] Shao D., Zhai P., Del Re D.P., Sciarretta S., Yabuta N., Nojima H., Lim D.S., Pan D., Sadoshima J. (2014). A functional interaction between Hippo-YAP signalling and FoxO1 mediates the oxidative stress response. Nat. Commun..

[49] Murakami M., Nakagawa M., Olson E.N., Nakagawa O. (2005). A WW domain protein TAZ is a critical coactivator for TBX5, a transcription factor implicated in Holt-Oram syndrome. Proc. Natl. Acad. Sci. USA.

[50] Rosenbluh J., Nijhawan D., Cox A.G., Li X., Neal J.T., Schafer E.J., Zack T.I., Wang X., Tsherniak A., Schinzel A.C. (2012). beta-Catenin-driven cancers require a YAP1 transcriptional complex for survival and tumorigenesis. Cell.

[51] Hong J.H., Hwang E.S., McManus M.T., Amsterdam A., Tian Y., Kalmukova R., Mueller E., Benjamin T., Spiegelman B.M., Sharp P.A. (2005). TAZ, a transcriptional modulator of mesenchymal stem cell differentiation. Science.

[52] Yagi R., Chen L.F., Shigesada K., Murakami Y., Ito Y. (1999). A WW domain-containing yes-associated protein (YAP) is a novel transcriptional co-activator. EMBO J..

[53] Cui C.B., Cooper L.F., Yang X., Karsenty G., Aukhil I. (2003). Transcriptional coactivation of bone-specific transcription factor Cbfa1 by TAZ. Mol. Cell. Biol..

[54] Levy D., Adamovich Y., Reuven N., Shaul Y. (2008). Yap1 phosphorylation by c-Abl is a critical step in selective activation of proapoptotic genes in response to DNA damage. Mol. Cell.

[55] Jiao S., Li C., Hao Q., Miao H., Zhang L., Li L., Zhou Z. (2017). VGLL4 targets a TCF4-TEAD4 complex to coregulate Wnt and Hippo signalling in colorectal cancer. Nat. Commun..

[56] Jiao S., Wang H., Shi Z., Dong A., Zhang W., Song X., He F., Wang Y., Zhang Z., Wang W. (2014). A peptide mimicking VGLL4 function acts as a YAP antagonist therapy against gastric cancer. Cancer Cell.

[57] Zhang W., Gao Y., Li P., Shi Z., Guo T., Li F., Han X., Feng Y., Zheng C., Wang Z. (2014). VGLL4 functions as a new tumor suppressor in lung cancer by negatively regulating the YAP-TEAD transcriptional complex. Cell Res..

[58] Su T., Ludwig M.Z., Xu J., Fehon R.G. (2017). Kibra and Merlin Activate the Hippo Pathway Spatially Distinct from and Independent of Expanded. Dev. Cell.

[59] Angus L., Moleirinho S., Herron L., Sinha A., Zhang X., Niestrata M., Dholakia K., Prystowsky M.B., Harvey K.F., Reynolds P.A. (2012). Willin/FRMD6 expression activates the Hippo signaling pathway kinases in mammals and antagonizes oncogenic YAP. Oncogene.

[60] Meng Z., Moroishi T., Mottier-Pavie V., Plouffe S.W., Hansen C.G., Hong A.W., Park H.W., Mo J.S., Lu W., Lu S. (2015). MAP4K family kinases act in parallel to MST1/2 to activate LATS1/2 in the Hippo pathway. Nat. Commun..

[61] Chen R., Xie R., Meng Z., Ma S., Guan K.L. (2019). STRIPAK integrates upstream signals to initiate the Hippo kinase cascade. Nat. Cell Biol..

[62] Bae S.J., Ni L., Luo X. (2020). STK25 suppresses Hippo signaling by regulating SAV1-STRIPAK antagonism. eLife.

[63] Khalafalla F.G., Greene S., Khan H., Ilves K., Monsanto M.M., Alvarez R., Chavarria M., Nguyen J., Norman B., Dembitsky W.P. (2017). P2Y2 Nucleotide Receptor Prompts Human Cardiac Progenitor Cell Activation by Modulating Hippo Signaling. Circ. Res..

[64] Boggiano J.C., Vanderzalm P.J., Fehon R.G. (2011). Tao-1 phosphorylates Hippo/MST kinases to regulate the Hippo-Salvador-Warts tumor suppressor pathway. Dev. Cell.

[65] Poon C.L., Lin J.I., Zhang X., Harvey K.F. (2011). The sterile 20-like kinase Tao-1 controls tissue growth by regulating the Salvador-Warts-Hippo pathway. Dev. Cell.

[66] Flinn M.A., Link B.A., O’Meara C.C. (2020). Upstream regulation of the Hippo-Yap pathway in cardiomyocyte regeneration. Semin. Cell Dev. Biol..

[67] Ragni C.V., Diguet N., Le Garrec J.F., Novotova M., Resende T.P., Pop S., Charon N., Guillemot L., Kitasato L., Badouel C. (2017). Amotl1 mediates sequestration of the Hippo effector Yap1 downstream of Fat4 to restrict heart growth. Nat. Commun..

[68] Yu F.X., Zhao B., Panupinthu N., Jewell J.L., Lian I., Wang L.H., Zhao J., Yuan H., Tumaneng K., Li H. (2012). Regulation of the Hippo-YAP pathway by G-protein-coupled receptor signaling. Cell.

[69] Luo J., Yu F.X. (2019). GPCR-Hippo Signaling in Cancer. Cells.

[70] Guo T., Lu Y., Li P., Yin M.X., Lv D., Zhang W., Wang H., Zhou Z., Ji H., Zhao Y. (2013). A novel partner of Scalloped regulates Hippo signaling via antagonizing Scalloped-Yorkie activity. Cell Res..

[71] Moses K.A., DeMayo F., Braun R.M., Reecy J.L., Schwartz R.J. (2001). Embryonic expression of an Nkx2-5/Cre gene using ROSA26 reporter mice. Genesis.

[72] Gan W., Dai X., Dai X., Xie J., Yin S., Zhu J., Wang C., Liu Y., Guo J., Wang M. (2020). LATS suppresses mTORC1 activity to directly coordinate Hippo and mTORC1 pathways in growth control. Nat. Cell Biol..

[73] Porrello E.R., Mahmoud A.I., Simpson E., Hill J.A., Richardson J.A., Olson E.N., Sadek H.A. (2011). Transient regenerative potential of the neonatal mouse heart. Science.

[74] Porrello E.R., Mahmoud A.I., Simpson E., Johnson B.A., Grinsfelder D., Canseco D., Mammen P.P., Rothermel B.A., Olson E.N., Sadek H.A. (2013). Regulation of neonatal and adult mammalian heart regeneration by the miR-15 family. Proc. Natl. Acad. Sci. USA.

[75] Zhao B., Wei X., Li W., Udan R.S., Yang Q., Kim J., Xie J., Ikenoue T., Yu J., Li L. (2007). Inactivation of YAP oncoprotein by the Hippo pathway is involved in cell contact inhibition and tissue growth control. Genes Dev..

[76] Hao Y., Chun A., Cheung K., Rashidi B., Yang X. (2008). Tumor suppressor LATS1 is a negative regulator of oncogene YAP. J. Biol. Chem..

[77] Dong J., Feldmann G., Huang J., Wu S., Zhang N., Comerford S.A., Gayyed M.F., Anders R.A., Maitra A., Pan D. (2007). Elucidation of a universal size-control mechanism in Drosophila and mammals. Cell.

[78] Schlegelmilch K., Mohseni M., Kirak O., Pruszak J., Rodriguez J.R., Zhou D., Kreger B.T., Vasioukhin V., Avruch J., Brummelkamp T.R. (2011). Yap1 acts downstream of alpha-catenin to control epidermal proliferation. Cell.

[79] Zhang X., Milton C.C., Humbert P.O., Harvey K.F. (2009). Transcriptional output of the Salvador/warts/hippo pathway is controlled in distinct fashions in Drosophila melanogaster and mammalian cell lines. Cancer Res..

[80] Jopling C., Sleep E., Raya M., Marti M., Raya A., Izpisua Belmonte J.C. (2010). Zebrafish heart regeneration occurs by cardiomyocyte dedifferentiation and proliferation. Nature.

[81] Kikuchi K., Holdway J.E., Werdich A.A., Anderson R.M., Fang Y., Egnaczyk G.F., Evans T., Macrae C.A., Stainier D.Y., Poss K.D. (2010). Primary contribution to zebrafish heart regeneration by gata4(+) cardiomyocytes. Nature.

[82] Han Y., Chen A., Umansky K.B., Oonk K.A., Choi W.Y., Dickson A.L., Ou J., Cigliola V., Yifa O., Cao J. (2019). Vitamin D Stimulates Cardiomyocyte Proliferation and Controls Organ Size and Regeneration in Zebrafish. Dev. Cell.

[83] Wang J., Cao J., Dickson A.L., Poss K.D. (2015). Epicardial regeneration is guided by cardiac outflow tract and Hedgehog signalling. Nature.

[84] Jopling C., Sune G., Morera C., Izpisua Belmonte J.C. (2012). p38alpha MAPK regulates myocardial regeneration in zebrafish. Cell Cycle.

[85] Wang J., Karra R., Dickson A.L., Poss K.D. (2013). Fibronectin is deposited by injury-activated epicardial cells and is necessary for zebrafish heart regeneration. Dev. Biol..

[86] Lai J.K.H., Collins M.M., Uribe V., Jimenez-Amilburu V., Gunther S., Maischein H.M., Stainier D.Y.R. (2018). The Hippo pathway effector Wwtr1 regulates cardiac wall maturation in zebrafish. Development.

[87] Dupont S., Morsut L., Aragona M., Enzo E., Giulitti S., Cordenonsi M., Zanconato F., Le Digabel J., Forcato M., Bicciato S. (2011). Role of YAP/TAZ in mechanotransduction. Nature.

[88] Wada K., Itoga K., Okano T., Yonemura S., Sasaki H. (2011). Hippo pathway regulation by cell morphology and stress fibers. Development.

[89] Aragona M., Panciera T., Manfrin A., Giulitti S., Michielin F., Elvassore N., Dupont S., Piccolo S. (2013). A mechanical checkpoint controls multicellular growth through YAP/TAZ regulation by actin-processing factors. Cell.

[90] Mosqueira D., Pagliari S., Uto K., Ebara M., Romanazzo S., Escobedo-Lucea C., Nakanishi J., Taniguchi A., Franzese O., Di Nardo P. (2014). Hippo pathway effectors control cardiac progenitor cell fate by acting as dynamic sensors of substrate mechanics and nanostructure. ACS Nano.

[91] Morikawa Y., Heallen T., Leach J., Xiao Y., Martin J.F. (2017). Dystrophin-glycoprotein complex sequesters Yap to inhibit cardiomyocyte proliferation. Nature.

[92] Bassat E., Mutlak Y.E., Genzelinakh A., Shadrin I.Y., Baruch Umansky K., Yifa O., Kain D., Rajchman D., Leach J., Riabov Bassat D. (2017). The extracellular matrix protein agrin promotes heart regeneration in mice. Nature.

[93] Apostolou E., Hochedlinger K. (2013). Chromatin dynamics during cellular reprogramming. Nature.

[94] Wolffe A.P., Guschin D. (2000). Review: Chromatin structural features and targets that regulate transcription. J. Struct. Biol..

[95] O’Meara C.C., Wamstad J.A., Gladstone R.A., Fomovsky G.M., Butty V.L., Shrikumar A., Gannon J.B., Boyer L.A., Lee R.T. (2015). Transcriptional reversion of cardiac myocyte fate during mammalian cardiac regeneration. Circ. Res..

[96] Sahara M., Santoro F., Chien K.R. (2015). Programming and reprogramming a human heart cell. EMBO J..

[97] Laflamme M.A., Murry C.E. (2011). Heart regeneration. Nature.

[98] Zhang R., Han P., Yang H., Ouyang K., Lee D., Lin Y.F., Ocorr K., Kang G., Chen J., Stainier D.Y. (2013). In vivo cardiac reprogramming contributes to zebrafish heart regeneration. Nature.

[99] Gregorieff A., Liu Y., Inanlou M.R., Khomchuk Y., Wrana J.L. (2015). Yap-dependent reprogramming of Lgr5(+) stem cells drives intestinal regeneration and cancer. Nature.

[100] Yimlamai D., Christodoulou C., Galli G.G., Yanger K., Pepe-Mooney B., Gurung B., Shrestha K., Cahan P., Stanger B.Z., Camargo F.D. (2014). Hippo pathway activity influences liver cell fate. Cell.

[101] Monroe T.O., Hill M.C., Morikawa Y., Leach J.P., Heallen T., Cao S., Krijger P.H.L., de Laat W., Wehrens X.H.T., Rodney G.G. (2019). YAP Partially Reprograms Chromatin Accessibility to Directly Induce Adult Cardiogenesis In Vivo. Dev. Cell.

[102] Gonzalez-Rosa J.M., Sharpe M., Field D., Soonpaa M.H., Field L.J., Burns C.E., Burns C.G. (2018). Myocardial Polyploidization Creates a Barrier to Heart Regeneration in Zebrafish. Dev. Cell.

[103] Kadow Z.A., Martin J.F. (2018). A Role for Ploidy in Heart Regeneration. Dev. Cell.

[104] Patterson M., Barske L., Van Handel B., Rau C.D., Gan P., Sharma A., Parikh S., Denholtz M., Huang Y., Yamaguchi Y. (2017). Frequency of mononuclear diploid cardiomyocytes underlies natural variation in heart regeneration. Nat. Genet..

[105] Del Re D.P., Matsuda T., Zhai P., Gao S., Clark G.J., Van Der Weyden L., Sadoshima J. (2010). Proapoptotic Rassf1A/Mst1 signaling in cardiac fibroblasts is protective against pressure overload in mice. J. Clin. Investig..

[106] Matsuda T., Zhai P., Sciarretta S., Zhang Y., Jeong J.I., Ikeda S., Park J., Hsu C.P., Tian B., Pan D. (2016). NF2 Activates Hippo Signaling and Promotes Ischemia/Reperfusion Injury in the Heart. Circ. Res..

[107] Yang Y., Del Re D.P., Nakano N., Sciarretta S., Zhai P., Park J., Sayed D., Shirakabe A., Matsushima S., Park Y. (2015). miR-206 Mediates YAP-Induced Cardiac Hypertrophy and Survival. Circ. Res..

[108] Tallquist M.D., Molkentin J.D. (2017). Redefining the identity of cardiac fibroblasts. Nat. Rev. Cardiol..

[109] Ameyar M., Wisniewska M., Weitzman J.B. (2003). A role for AP-1 in apoptosis: The case for and against. Biochimie.

[110] Xin M., Olson E.N., Bassel-Duby R. (2013). Mending broken hearts: Cardiac development as a basis for adult heart regeneration and repair. Nat. Rev. Mol. Cell Biol..

[111] Norton G.R., Woodiwiss A.J., Gaasch W.H., Mela T., Chung E.S., Aurigemma G.P., Meyer T.E. (2002). Heart failure in pressure overload hypertrophy. The relative roles of ventricular remodeling and myocardial dysfunction. J. Am. Coll. Cardiol..

[112] Ikeda S., Mizushima W., Sciarretta S., Abdellatif M., Zhai P., Mukai R., Fefelova N., Oka S.I., Nakamura M., Del Re D.P. (2019). Hippo Deficiency Leads to Cardiac Dysfunction Accompanied by Cardiomyocyte Dedifferentiation During Pressure Overload. Circ. Res..

[113] Nakamura M., Sadoshima J. (2019). Cardiomyopathy in obesity, insulin resistance and diabetes. J. Physiol..

[114] Seferovic P.M., Paulus W.J. (2015). Clinical diabetic cardiomyopathy: A two-faced disease with restrictive and dilated phenotypes. Eur. Heart J..

[115] Triastuti E., Nugroho A.B., Zi M., Prehar S., Kohar Y.S., Bui T.A., Stafford N., Cartwright E.J., Abraham S., Oceandy D. (2019). Pharmacological inhibition of Hippo pathway, with the novel kinase inhibitor XMU-MP-1, protects the heart against adverse effects during pressure overload. Br. J. Pharmacol..

[116] Epelman S., Liu P.P., Mann D.L. (2015). Role of innate and adaptive immune mechanisms in cardiac injury and repair. Nat. Rev. Immunol..

[117] Lai S.L., Marin-Juez R., Stainier D.Y.R. (2019). Immune responses in cardiac repair and regeneration: A comparative point of view. Cell. Mol. Life Sci. CMLS.

[118] Aurora A.B., Porrello E.R., Tan W., Mahmoud A.I., Hill J.A., Bassel-Duby R., Sadek H.A., Olson E.N. (2014). Macrophages are required for neonatal heart regeneration. J. Clin. Investig..

[119] Coggins M., Rosenzweig A. (2012). The fire within: Cardiac inflammatory signaling in health and disease. Circ. Res..

[120] Morimoto H., Takahashi M., Izawa A., Ise H., Hongo M., Kolattukudy P.E., Ikeda U. (2006). Cardiac overexpression of monocyte chemoattractant protein-1 in transgenic mice prevents cardiac dysfunction and remodeling after myocardial infarction. Circ. Res..

[121] Vagnozzi R.J., Maillet M., Sargent M.A., Khalil H., Johansen A.K.Z., Schwanekamp J.A., York A.J., Huang V., Nahrendorf M., Sadayappan S. (2020). An acute immune response underlies the benefit of cardiac stem cell therapy. Nature.

[122] Cai C.L., Molkentin J.D. (2017). The Elusive Progenitor Cell in Cardiac Regeneration: Slip Slidin’ Away. Circ. Res..

[123] Maliken B.D., Molkentin J.D. (2018). Undeniable Evidence That the Adult Mammalian Heart Lacks an Endogenous Regenerative Stem Cell. Circulation.

[124] Pillemer L., Blum L., Pensky J., Lepow I.H. (1953). The requirement for magnesium ions in the inactivation of the third component of human complement (C’3) by insoluble residues of yeast cells (zymosan). J. Immunol..

[125] Ramjee V., Li D., Manderfield L.J., Liu F., Engleka K.A., Aghajanian H., Rodell C.B., Lu W., Ho V., Wang T. (2017). Epicardial YAP/TAZ orchestrate an immunosuppressive response following myocardial infarction. J. Clin. Investig..

[126] Hofmann U., Beyersdorf N., Weirather J., Podolskaya A., Bauersachs J., Ertl G., Kerkau T., Frantz S. (2012). Activation of CD4+ T lymphocytes improves wound healing and survival after experimental myocardial infarction in mice. Circulation.

[127] Nahrendorf M., Swirski F.K. (2014). Regulating repair: Regulatory T cells in myocardial infarction. Circ. Res..

[128] Weirather J., Hofmann U.D., Beyersdorf N., Ramos G.C., Vogel B., Frey A., Ertl G., Kerkau T., Frantz S. (2014). Foxp3+ CD4+ T cells improve healing after myocardial infarction by modulating monocyte/macrophage differentiation. Circ. Res..

[129] Zacchigna S., Martinelli V., Moimas S., Colliva A., Anzini M., Nordio A., Costa A., Rehman M., Vodret S., Pierro C. (2018). Paracrine effect of regulatory T cells promotes cardiomyocyte proliferation during pregnancy and after myocardial infarction. Nat. Commun..

[130] Hulsmans M., Clauss S., Xiao L., Aguirre A.D., King K.R., Hanley A., Hucker W.J., Wulfers E.M., Seemann G., Courties G. (2017). Macrophages Facilitate Electrical Conduction in the Heart. Cell.

[131] King K.R., Aguirre A.D., Ye Y.X., Sun Y., Roh J.D., Ng R.P., Kohler R.H., Arlauckas S.P., Iwamoto Y., Savol A. (2017). IRF3 and type I interferons fuel a fatal response to myocardial infarction. Nat. Med..

[132] Francisco J., Byun J., Zhang Y., Kalloo O.B., Mizushima W., Oka S., Zhai P., Sadoshima J., Del Re D.P. (2019). The tumor suppressor RASSF1A modulates inflammation and injury in the reperfused murine myocardium. J. Biol. Chem..

[133] Bertero A., Murry C.E. (2018). Hallmarks of cardiac regeneration. Nat. Rev. Cardiol..

[134] Moya I.M., Halder G. (2019). Hippo-YAP/TAZ signalling in organ regeneration and regenerative medicine. Nat. Rev. Mol. Cell Biol..

[135] Chen X., Li Y., Luo J., Hou N. (2020). Molecular Mechanism of Hippo-YAP1/TAZ Pathway in Heart Development, Disease, and Regeneration. Front. Physiol..

[136] Huang J., Wu S., Barrera J., Matthews K., Pan D. (2005). The Hippo signaling pathway coordinately regulates cell proliferation and apoptosis by inactivating Yorkie, the Drosophila Homolog of YAP. Cell.

[137] Overholtzer M., Zhang J., Smolen G.A., Muir B., Li W., Sgroi D.C., Deng C.X., Brugge J.S., Haber D.A. (2006). Transforming properties of YAP, a candidate oncogene on the chromosome 11q22 amplicon. Proc. Natl. Acad. Sci. USA.

